# What Have We Learnt About the Treatment of Juvenile-Onset Systemic Lupus Erythematous Since Development of the SHARE Recommendations 2012?

**DOI:** 10.3389/fped.2022.884634

**Published:** 2022-04-14

**Authors:** Kathy L. Gallagher, Pallavi Patel, Michael W. Beresford, Eve Mary Dorothy Smith

**Affiliations:** ^1^Paediatric Rheumatology, Great Ormond Street Hospital for Children NHS Foundation Trust, London, United Kingdom; ^2^Department of Public Health, Liverpool City Council, Liverpool, United Kingdom; ^3^Institute of Life Course and Medical Science, University of Liverpool, Liverpool, United Kingdom; ^4^Department of Paediatric Rheumatology, Alder Hey Children's NHS Foundation Trust, Liverpool, United Kingdom

**Keywords:** childhood-onset systemic lupus erythematous, juvenile-onset systemic lupus erythematous, lupus nephritis, antiphospholipid syndrome, treatment, biologics, pediatric rheumatology

## Abstract

**Introduction:**

Juvenile-onset systemic lupus erythematous (JSLE) is a rare multisystem autoimmune disorder. In 2012, the Single Hub and Access point for pediatric Rheumatology in Europe (SHARE) initiative developed recommendations for the diagnosis/management of JSLE, lupus nephritis (LN) and childhood-onset anti-phospholipid syndrome (APS). These recommendations were based upon available evidence informing international expert consensus meetings.

**Objective:**

To review new evidence published since 2012 relating to the management of JSLE, LN and APS in children, since the original literature searches informing the SHARE recommendations were performed.

**Method:**

MEDLINE, EMBASE and CINAHL were systematically searched for relevant literature (2012-2021) using the following criteria: (1) English language studies; (2) original research studies regarding management of JSLE, LN, APS in children; (3) adult studies with 3 or more patients <18-years old, or where the lower limit of age range ≤16-years and the mean/median age is ≤30-years; (4) randomized controlled trials (RCTs), cohort studies, case control studies, observational studies, case-series with >3 patients. Three reviewers independently screened all titles/abstracts against predefined inclusion/exclusion criteria. All relevant manuscripts were reviewed independently by at least two reviewers. Data extraction, assessment of the level of evidence/methodological quality of the manuscripts was undertaken in-line with the original SHARE processes. Specific PUBMED literature searches were also performed to identify new evidence relating to each existing SHARE treatment recommendation.

**Results:**

Six publications met the inclusion/exclusion criteria for JSLE: three RCTs, one feasibility trial, one case series. For LN, 16 publications met the inclusion/exclusion criteria: eight randomized trials, three open label prospective clinical trials, five observational/cohort studies. For APS, no publications met the inclusion criteria. The study with the highest evidence was an RCT comparing belimumab vs. placebo, including 93 JSLE patients. Whilst the primary-endpoint was not met, a significantly higher proportion of belimumab-treated patients met the PRINTO/ACR cSLE response to therapy criteria. New evidence specifically addressing each SHARE recommendation remains limited.

**Conclusion:**

Since the original SHARE literature searches, undertaken >10-years ago, the main advance in JSLE treatment evidence relates to belimumab. Additional studies are urgently needed to test new/existing agents, and assess their long-term safety profile in JSLE, to facilitate evidence-based practice.

## Introduction

Juvenile-onset systemic lupus erythematous (JSLE) is a rare multisystem autoimmune disorder with significant associated morbidity and potentially life-threatening complications. It has an estimated of incidence of 0.3–0.9 per 100,000 children-years, with a prevalence of 1.89 to 25.7 per 100,000 children worldwide (1). Lupus nephritis (LN) occurs in 50–80% of patients with JSLE (2, 3). Childhood-onset antiphospholipid syndrome (APS) is also associated with JSLE although its prevalence is very low (4). Early recognition and treatment of these manifestations of JSLE is essential for prevention of potential morbidity and mortality.

In 2012, the Single Hub and Access point for pediatric rheumatology (SHARE) in Europe developed recommendations for the management of JSLE including LN and also APS (1, 2, 5). The aim of SHARE was to produce international, evidence-based consensus recommendations for the diagnosis, investigation, and management of JSLE. This was undertaken to address the variable practice observed in management of JSLE, resulting primarily from the lack of robust research to inform evidence-based practice (6). The first step taken in SHARE was to perform systematic literatures searches to inform discussions of a Europe-wide panel of pediatric rheumatologists (with representation from pediatric nephrology) during international expert consensus meetings to agree the recommendations. SHARE developed five recommendations for treatment of JSLE in general, 20 for LN, and eight for pediatric APS (1).

The TARGET LUPUS research programme has been established in order to develop a “treat to target” (T2T) approach for JSLE, with the aim of improving outcomes through implementation of a structured approach to treatment (7, 8). T2T has been successfully used for the management of chronic diseases such as rheumatoid arthritis and Juvenile Idiopathic arthritis (9–11). Understanding of the evidence base underlying treatment decisions in JSLE is essential for the development of protocol driven therapeutic strategies for use within a future T2T study.

The aim of the current study was to review all new evidence relating to the management of JSLE, LN and childhood-onset APS since the original SHARE comprehensive review was undertaken, to help inform development of T2T organ domain driven therapeutic strategies.

## Methodology

### Search Strategy

Relevant papers were identified in MEDLINE, EMBASE and CINAHL bibliographic databases following the initial SHARE methodology. Studies were eligible for inclusion if they fulfilled the following criteria: (a) published in English language; (b) from 2012–September 2021; (c) original research studies regarding management of JSLE, LN and/or childhood-onset APS; and either (d) pediatric studies or e) adult SLE studies meeting the following criteria were included: (i) 3 or more patients <18 years of age, or (ii) when lower limit of age range ≤16 years: include if study has more than 15 patients AND a mean/median age ≤30 years.

Publications were excluded for the following reasons: (a) publications on other diseases (e.g., vasculitis, adult SLE alone); (b) with a focus on aspects other than management; (c) case report with <3 patients; (d) conference abstracts only or full text unavailable; e) reviews; (f) adult studies not fulfilling age criteria; (g) non-human data; and h) not published in English. Further literature searches were performed to assess if there was any specific new evidence within the pediatric or adult SLE literature related to each of the existing SHARE treatment recommendations for JSLE in general, LN and/or APS.

### Screening Criteria

Three reviewers (PP, KG, ES) independently screened all publications (titles, abstracts) according to the studies predefined inclusion/exclusion criteria. All relevant publications were retrieved and reviewed by at least two of the three reviewers. For publications where the age criteria were unclear, corresponding authors were contacted. Of the 10 authors contacted, three provided further information.

### Data Collection

Data extraction was performed using the original SHARE data extraction sheet for treatment (see Supplemental File 1). The extraction sheet included: general study information, study population characteristics, study methods, results, conclusions/discussions, validity assessment and category of evidence. Data was collected by one author and reviewed by two more authors independently. For publications where there was any uncertainty, a face-to-face discussion was held.

## Results

### Literature Searches

**Figure 1:**
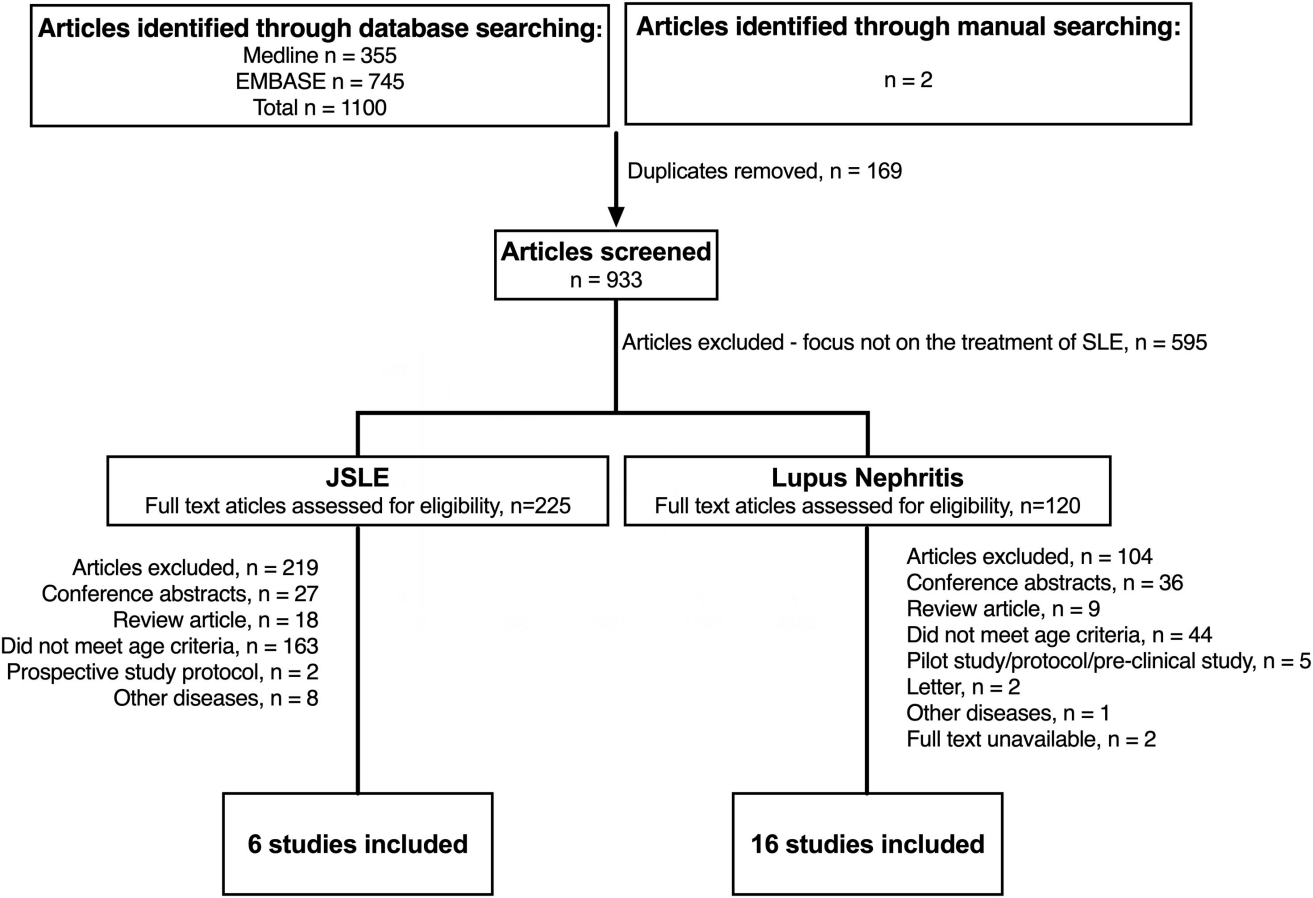
Identification of studies evaluating treatment of JSLE in general and lupus nephritis since 2012. N, number of patients; JSLE, Juvenile Systemic Lupus Erythematosus; SLE, Systemic Lupus Erythematosus.

**Figure 2:**
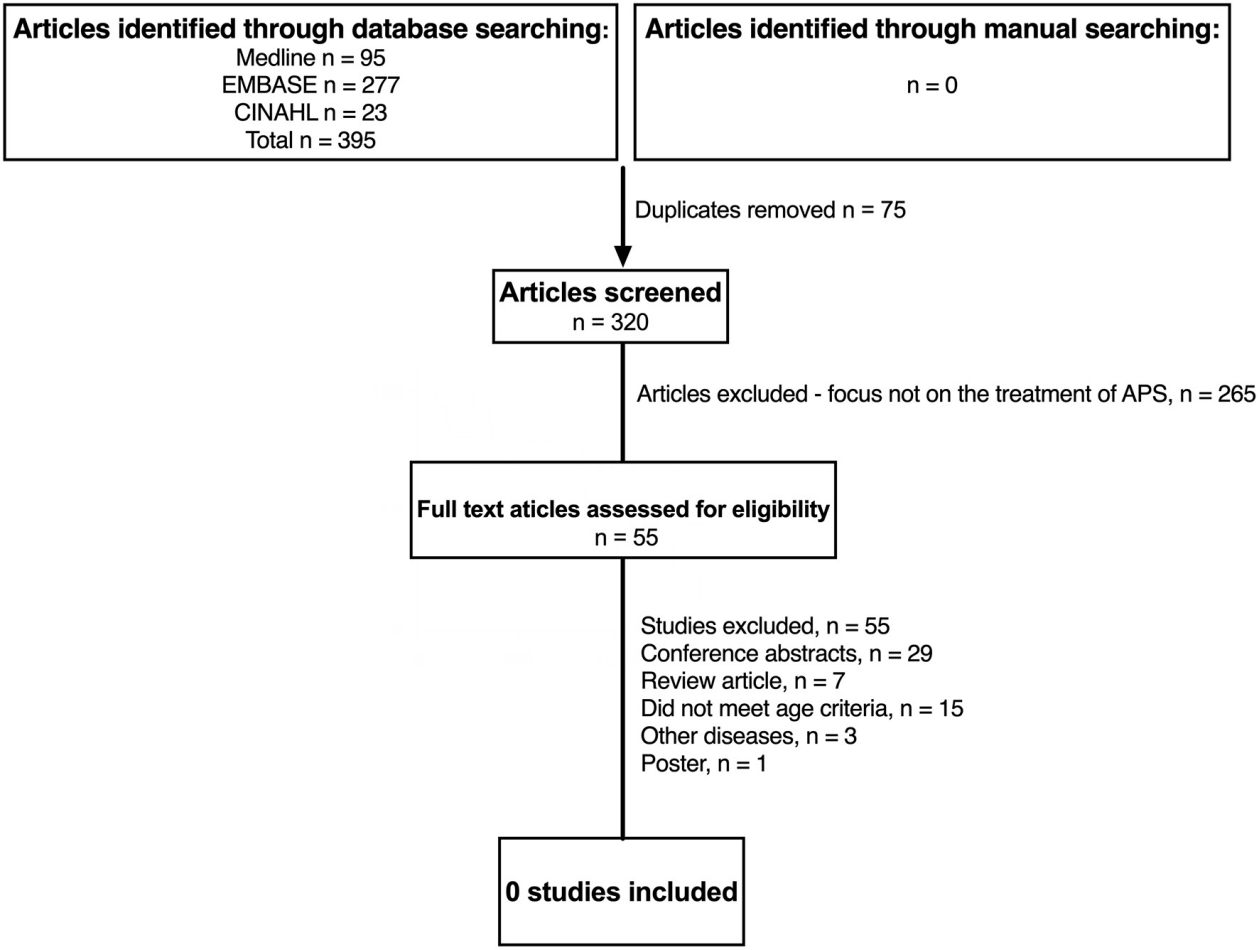
Identification of studies evaluating treatment of childhood antiphospholipid syndrome since 2012. N, number of patients; APS, anti-phospholipid syndrome; JSLE, Juvenile Systemic Lupus Erythematosus; SLE, Systemic Lupus Erythematosus.

Figure 1 summaries the results of the literature search in JSLE and LN. Both searches contained the term “lupus” (lupus nephritis AND juvenile systemic lupus erythematosus), therefore the same publications were captured by each search (*n* = 1,100). For JSLE in general, after screening of the titles and abstracts, 225 publications were identified as relevant to the management of JSLE. Full text publications were then assessed against the inclusion/exclusion criteria, with 6 publications identified as meeting the full criteria (two of which related to the same trial). For LN, after screening of the titles and abstracts, 118 publications were identified as relevant to the management of LN. Of those, 16 publications met the full inclusion/exclusion criteria. Figure 2 summaries the results of the literature search in APS. The literature search produced 395 publications. After screening of the titles and abstracts, 55 publications were identified as relevant to the management of APS but no publications met the full inclusion/exclusion criteria.

#### Evidence Relating to Management of JSLE in General

Table 1 summarizes the six publications relating to the management of JSLE in general, two relating to one trial on prevention of atherosclerosis, one on immunosuppressive treatment, one on prevention and treatment of osteopenia, and the last on interventions to improve health related quality of life (HRQOL).

**Table 1:** Summary of pediatric lupus treatment studies from 2012.

**Study**	**Patients**	**Outcome measures**	**Main result(s)**	**Safety outcomes**	**Level of evidence**
Safety and efficacy of intravenous belimumab in children with systemic lupus erythematosus: results from a randomized, placebo-controlled trial Brunner et al. (12)	Total 93	Primary endpoint: SLE Responder Index 4 (SRI4) response rate at Week 52 Secondary endpoints included: proportion of patients meeting the (PRINTO/ACR) jSLE criteria for response to therapy, Parent Global Assessment of patient overall well-being, PedsQL, proteinuria	No statistically significant difference in primary endpoint (although numerically higher proportion of belimumab patients achieved this). Significantly higher proportion of belimumab patients achieved both the PRINTO/ACR 30 [52.8% vs. 27.5%; OR 2.92 (95% CI 1.19 to 7.17)] and PRINTO/ACR 50 [60.4% vs. 35.0%; OR 2.74 (95% CI 1.15 to 6.54)] responses	Similar incidence of adverse events in treatment group (79.2%) compared to placebo group (82.5%)	1B
Use of Atorvastatin in Systemic Lupus Erythematosus in Children and Adolescents Schanberg et al. (13)	Total 221 Atorvastatin group 113, placebo 108bo group−108	Mean-mean common carotid intima-media thickening (CIMT) measured by ultrasound	No significant difference observed between atorvastatin and placebo	The occurrence of serious adverse events and predefined safety events (muscle, liver, and neurotoxicity) did not differ between the treatment groups	1B
Secondary analysis of APPLE study suggests atorvastatin may reduce atherosclerosis progression in pubertal lupus patients with higher C reactive protein Ardoin et al. (14) [Secondary analysis of study carried out by Schanberg et al. (13)]	Total 221 Atorvastatin 113, placebo 108	Mean-mean common carotid intima-media (CIMT) thickening measured by ultrasound. Three arterial segments measured, with a total of 12 measurement sites.	Pubertal patient subgroup: -Significant reduction in CIMT progression and increase in HRQOL in atorvastatin treated patients High CRP subgroup: -Lower CIMT progression in two carotid artery segments with atorvastatin, increased HRQOL Combined pubertal and high CRP subgroup (vs. all other patients): -Significantly less CIMT progression in 5 of 12 CIMT measurement sites	As above	1B
The prevention and treatment of glucocorticoid-induced osteopenia in juvenile rheumatic disease: A randomized double-blind controlled trial Rooney et al. (15)	Total 217 76 SLE patients Risedronate 69 (24 SLE) Alfacalcidol 71 (21 SLE) Placebo 77 (31 SLE)	Change in lumbar spine BMD z score measured by dual energy x-ray absorptiometry at 1 year.	Risedronate group demonstrated increased lumbar spine BMD z-score as well as Total Body (Less Head) Area l-bone mineral density z-score compared to placebo (*p <* 0.001) and compared to alfacalcidol (*p <* 0.001).	No significant differences in fracture frequency, adverse or serious adverse reactions were observed between the groups.	1B
The Health Education for Lupus Patients Study: A Randomized Controlled Cognitive-Behavioral Intervention Targeting Psychosocial Adjustment and Quality of Life in Adolescent Females with Systemic Lupus Erythematosus Brown et al. (16)	Total 53 CBT−27 Education only - 10 Control - 16	The McGill Pain Questionnaire. The Behavior Assessment System for Children (BASC). Positive and Negative Affect Schedule-Extended Version. The Self-Perception Profile for Adolescents (SPPA). The Multidimensional Health Locus of Control Scales. PedsQL.	No significant differences in outcomes among the CBT, education-only and control groups. Secondary analysis showed increased coping skills in the CBT group compared to education-only and control groups.	n/a	1B
Rituximab use in pediatric lupus anticoagulant hypo-prothrombinemia syndrome–report of three cases and review of the literature Gedik et al. (17)	Total 3	Complete resolution defined as no further bleeding diathesis/thrombotic events and normal factor II level Partial resolution defined as improved or resolved bleeding diathesis/thrombotic events and improved factor II level	Partial resolution in two patients, complete resolution in one patient	Not discussed	3

*APPLE, Atherosclerosis Prevention in Pediatric Lupus Erythematosus trial; HRQOL, Health Related Quality of life; BMD, bone mineral density; CBT, cognitive behavioral therapy; PedsQL, Pediatric Quality of Life Inventory; SR14, SLE responder index 4; PRINTO, Pediatric Rheumatology International Trials Organization; ACR, American College of Rheumatology*.

Belimumab, a monoclonal antibody targeting the B-lymphocyte stimulator (BLYS), has previously been approved for use in active adult-onset SLE patients with elevated anti-dsDNA titres and/or low complement levels (18, 19). This was following *post hoc* analysis of the original trial data demonstrating a better response to belimumab in this sub-group of active adult SLE patients. More recently the PLUTO study, an RCT comparing intravenous belimumab (10 mg/kg) plus standard JSLE therapy to placebo in 93 patients with active JSLE, demonstrating that a numerically higher proportion of patients receiving belimumab met the primary endpoint of SLE Responder Index 4 [SRI4; 52.8 vs. 43.6%; OR 1.49 (95% CI 0.64 to 3.46)] (12). As the confidence interval crossed 1, this did not meet statistical significance. The SRI4 was used as a primary outcome measure for comparability with the original adult-onset SLE Belimumab trial.

The major secondary endpoint was the proportion of patients meeting the Pediatric Rheumatology International Trials Organization / American College of Rheumatology (PRINTO/ACR) JSLE criteria for response to therapy (20). A significantly higher proportion patients treated with belimumab achieved both the PRINTO/ACR 30 [52.8 vs. 27.5%; OR 2.92 (95% CI 1.19 to 7.17)] and PRINTO/ACR 50 [60.4 vs. 35.0%; OR 2.74 (95% CI 1.15 to 6.54)] responses (12). These results have led to both the European Medicines Agency (EMA) and Food and Drug Administration (FDA) approving use of Belimumab in JSLE (21, 22).

The APPLE (Atherosclerosis Prevention in Pediatric Lupus Erythematosus) study (13) demonstrated no significant benefit from atorvastatin in reducing progression of atherosclerosis during three-years of treatment, as measured by carotid intima-media thickness (CIMT) in 113 patients with SLE aged 10–21 years. The atorvastatin was well tolerated over 3-years. The occurrence of serious adverse events and predefined safety events did not differ between the treatment groups. Secondary analyses from this study, within a subsequent paper, suggested that there may be a benefit for statin therapy in pubertal SLE patients with a raised CRP, with this subgroup showing reduced CIMT progression (14).

A randomized double-blind placebo-controlled trial comparing 1 year of treatment with risedronate or alfacalcidol with placebo, for glucocorticoid-induced osteopenia in juvenile rheumatic disease published its results in 2019. 35% of patients (76/217) had JSLE. It demonstrated that risedronate significantly increased bone mass in patients with low bone mass (lumbar spine bone mineral density z score +0.274, 95% CI (0.061, 0.487) (*p* < 0.001) in risedronate treated patients). There was no significant difference between the alfacalcidol and placebo groups (15). The study concluded that risedronate should be considered for children receiving steroid treatment to reduce fracture risk.

The Health Education for Lupus Feasibility Trial explored psychosocial adjustment and HRQOL in female adolescent SLE patients (16). Patients received either: (a) cognitive behavioral therapy (CBT), (b) education only or (c) no intervention (control). While there were no statistically significant differences among the three treatment arms, secondary analyses suggested increased coping skills in the group who received CBT. This is one of very few studies exploring non-pharmacological management of JSLE, highlighting the need for further studies exploring other forms of support and management in JSLE.

A case series of three JSLE patients suggested rituximab may be a useful steroid-sparing treatment for lupus anticoagulant hypoprothrombinaemia syndrome. This rare manifestation of SLE is caused by presence of lupus anticoagulant and factor II deficiency, increasing risk of serious bleeding and thrombosis. In 2/3 cases there was partial resolution of their lupus anticoagulant hypoprothombinaemia syndrome features and complete resolution in the remaining patient following rituximab treatment (17).

### New Evidence Relating to General JSLE SHARE Treatment Recommendations

The SHARE recommendations state “*all children with lupus should be on hydroxychloroquine routinely”* (1). No new pediatric studies relating to this recommendation could be identified. In adult-SLE, a Canadian cohort study reported that “more consistent” use of antimalarials in the first 5-years following SLE diagnosis (defined as patient reported antimalarial agent use >60% of the time) was associated with reduced risk of Systemic Lupus International Collaborating Clinics/American College of Rheumatology Damage Index (SLICC-SDI) score defined damage, increased achievement of low disease activity (defined as a clinical-SLEDAI-2K score of ≤2, not including serology) and reduced cumulative glucocorticoid dose after 5-years of follow-up.

A study examining long-term outcomes in Dutch adults with JSLE has also shown current hydroxychloroquine monotherapy to be associated with absence of SLICC-SDI defined damage (23). A large population based study using hospital episode statistics and national death certificates (from 1987 to 2012) has demonstrated that hydroxychloroquine use is associated with a 45% reduction in the hazards of mortality in adult-SLE (24). Collectively these more recent adult-SLE studies support this SHARE recommendation.

The increasing evidence regarding long term side effects of hydroxychloroquine should however be considered. Due to advances in eye screening, hydroxychloroquine retinopathy has been found to be more common than previously thought, with a 2014 study showing a prevalence of 7.5% in adult-SLE patients taking hydroxychloroquine for a minimum of 5-years. A total daily dose of >5 mg/kg (using actual body weight) was found to be associated with increased risk. Renal impairment and tamoxifen therapy also increased the risk of retinopathy (25). The SHARE recommendations currently advise that yearly eye screening should be “considered” in children taking hydroxychloroquine (1). The UK Royal College of Ophthalmologists guideline (for adult use) states that when long-term hydroxychloroquine treatment is planned, patients should receive a baseline examination (within 12-months), followed by annual screening from year 5 of treatment onwards. In patients with additional risk factors for retinopathy (e.g., Tamoxifen use, impaired renal function (estimated glomerular filtration rate of <60 ml/min/1.73 m^2^), hydroxychloroquine dose >5 mg/kg/day) annual monitoring from baseline is recommended. Despite a lack of evidence in patients <18-years, these guidelines advise that these patients on long term hydroxychloroquine should also be referred for monitoring as per the adult criteria (26).

SHARE advises that “*in all decisions of treatment change or modification, compliance should be actively checked”*. Patient compliance is known to be a challenge in JSLE, with a previous study showing only 32% of adolescents and young adults with SLE to be compliant with hydroxychloroquine (27), and a further study demonstrating 43–75% of adult-SLE patients to be non-compliant with hydroxychloroquine (28). Despite this, drug levels are not routinely monitored in clinical practice. The 2012 PLUS study found that tailoring hydroxychloroquine dose to a target therapeutic blood concentration did not reduce the frequency of SLE flares. However, hydroxychloroquine blood levels increased spontaneously between study inclusion and randomization, suggesting improved adherence to hydroxychloroquine treatment in all patients, likely in response to the information that was sent to patients about the study. This study therefore concluded that despite the trial not meeting its primary endpoint, routine measurement of hydroxychloroquine levels may improve patient adherence to treatment (29). A recent meta-analysis found a good association between whole-blood hydroxychloroquine levels and reported non-adherence (30).

In 2018, a web-based education and a social media intervention was shown to significantly improve adherence to medications in adolescents and young adults with SLE (31). Within this study, self-reported medication adherence was significantly higher than objectively measured indices of adherence (e.g., the medication possession ratio), highlighting the need for objective measures of adherence, such as blood levels. Novel approaches such as social media interventions may help empower patients to manage their own medications effectively (31).

In relation to corticosteroid therapy, the SHARE recommendations advise that “*when it is not possible to taper the prednisone dose, a DMARD should be added to the therapy”*. No new original pediatric research was found relating to this recommendation. However, the 2019 update to the EULAR recommendations for SLE also advise addition of methotrexate, azathioprine, or mycophenolate mofetil (MMF) in patients whose symptoms are not controlled with corticosteroids and hydroxychloroquine. Cyclophosphamide is suggested for severe organ threatening or life-threatening SLE or for patients who do not respond to other immunosuppressive agents. These recommendations advise that belimumab is used for patients with frequent relapses or those not able to taper steroid dose despite the above standard of care. Consideration of rituximab is suggested for organ-threatening disease refractory to standard immunosuppressive agents or where these are contraindicated or not tolerated (32).

SHARE advises that “*in mild/moderate hematological involvement: when haemolysis is present and Hb is lower than normal, a DMARD should be added to the therapy”*. In 2015, a retrospective cohort study assessed 24 JSLE patients treated with rituximab for refractory cytopenias, 19 of whom had haemolytic anemia. Overall, 96% of patients showed complete response after the first course of rituximab (defined as Hb >120 g/L for haemolytic anemia and platelet count >100 x 10^9^/L for patients with thrombocytopenia). The median time to complete response for patients with haemolytic anemia was 85 days (33). A Turkish study examining hematological involvement in JSLE also found benefit from rituximab in cases of haemolytic anemia resistant to steroid and intravenous immunoglobulin (IVIG) treatment (34).

The SHARE recommendations state that “*if rituximab is required, the recommended dose is either 750 mg/m*^2^*/dose (up to a maximum of 1 g) at day 1 and day 15, or 375 mg/m*^2^*/dose once a week for four doses”*. No new pediatric research was identified examining dose regimes for rituximab. In 2014, a UK study of rituximab use in patients with JSLE over a 10-year period (2003–2013) of 63 patients, all received a dose of 750 mg/m^2^/dose ~2-weeks apart (35). The new evidence relating to general JSLE SHARE treatment recommendations is summarized in Supplemental Table 1.

### New Evidence Relating to Neuropsychiatric JSLE SHARE Treatment Recommendations

JSLE SHARE treatment recommendations also included those directed toward neuropsychiatric manifestations (1). They recommend that “*When neuropsychiatric manifestations are caused by an immune or inflammatory process and non-SLE-related causes are excluded, corticosteroids and immunosuppressive therapy are indicated”*. Limited new evidence could be found relating to this recommendation in JSLE. A retrospective study of 144 children with autoimmune and inflammatory disorders of the central nervous system (CNS, 18/144 with NP-SLE) treated with rituximab demonstrated “definite” clinician-defined improvement with rituximab in 5/18 patients, “probable” in 7/18, “possible” in 5/18 and “no improvement” in 1/18 patients (36). A 2013 study of Saudi children included two cases of lupus cerebritis which improved with combined rituximab and cyclophosphamide treatment (37). A Chinese study of 20 children with SLE reported that in 10/20 (50%) cases, delirium and cognitive disorders improved after one-month of rituximab treatment (38). An Indian study of 88 adult patients with NP-SLE treated with MMF and deflazacort showed complete response (defined as complete resolution of initial neuropsychiatric signs and symptoms) in 83.9% of patients at 1-year follow up, and in 92.3% of patients at last follow up (median 33-months) (39).

SHARE also recommended that “*antiepileptic drugs are usually not necessary after a single seizure in the absence of MRI lesions and definite epileptic abnormalities on EEG following recovery from the seizure. Long-term antiepileptic therapy should be considered for recurrent seizures”* (2). No new evidence could be found relating to this recommendation. Overall, the evidence base for NP-SLE treatment in children is minimal and further studies are needed. The EULAR adult-SLE guidelines for NP-SLE are in-keeping with the SHARE recommendations, namely that “*Treatment of SLE-related neuropsychiatric disease includes glucocorticoids/immunosuppressive agents for manifestations considered to reflect an inflammatory process”* (32). The final SHARE recommendation is that “*there is a need for pediatric NP-cSLE research regarding treatment”* (2), and this clearly continues to be the case. New evidence relating to neuropsychiatric JSLE SHARE treatment recommendations is summarized in Supplemental Table 1.

### Evidence Relating to Management of LN in JSLE

Sixteen studies relating to management of LN in JSLE were identified: two pediatric studies, one adolescent study and 13 adult studies including JSLE patients. Tables 2–**4** summarizes these studies, and the key findings are discussed below.

**Table 2:** Summary of pediatric lupus nephritis treatment studies from 2012 including cyclophosphamide.

**Study**	**Patients**	**Treatment**	**Outcome measures**	**Main result(s)**	**Safety outcomes**	**Level of evidence**
Outcomes following mycophenolate mofetil vs. cyclophosphamide induction treatment for proliferative juvenile-onset lupus nephritis. Smith et al. (40) Study type: Cohort study from data collected in UK JSLE Cohort study Type of LN: renal biopsy result demonstrating ISN/RPS class III or IV	Total: 51 Pediatric study MMF induction 34 CYC 17 Age at biopsy (years) MMF 13.1 (11.2–15.0) CYC 13.6 (12.8–15.6)	Induction: MMF (follows the Euro-Lupus Nephritis Trial protocol) or IV CYC (500-1000mg/m2/day every 4 weeks for a total of 4–6 months). Concomitant corticosteroid treatment was also documented (oral prednisolone, intravenous methylprednisolone or both).	Descriptive study comparing disease activity scores (renal BILAG scores), laboratory parameters (urine albumin / creatinine ratio, serum creatinine, ESR, anti-dsDNA antibody, C3 levels), physician global scores, time to achievement of inactive LN and subsequent flare at 4–8, 10–14 months post LN induction treatment initiation and last follow-up. Standardized damage score (SLICC-SDI) compared at 10-14 months and last follow-up.	34/51 (67%) received MMF, and 17/51 (33%) received IV CYC induction treatment. No significant differences were identified at 4–8 and 10–14 months post-renal biopsy and last follow-up, in terms of renal BILAG scores, urine albumin/creatinine ratio, serum creatinine, ESR, anti-dsDNA antibody, C3 levels and patient/physician global scores (all p>0.05). SLICC-SDI score did not differ between treatment groups at 10-14 months or last follow-up. Inactive LN attained 262 (141–390) days after MMF treatment, and 151 (117–305) days following IV CYC (*p =* 0.17). Time to renal flare was 451 (157–1266) days for MMF, and 343 (198–635) days for IV CYC (*p =* 0.47).	Not assessed	3
Low dose mycophenolate mofetil vs. cyclophosphamide in the induction therapy of lupus nephritis in Nepalese population: a randomized control trial. Sedhain et al. (41) Study type*:* Randomized controlled trial Type of LN: biopsy proven class III, IV, V, III + V or IV + V based on ISN/RPS.	Adult study including some JSLE patients Total 42 M 5 F 37 Age range: 13–68 years Age (years): CYC 24.76 +/- 11.6 MMF 27.24 +/- 9.34	*Induction* MMF or CYC in those with proliferative lupus nephritis Other drugs: Prednisolone Hydroxychloroquine For hypertension, ACE/ARB	Primary end point: Decrease in proteinuria – reduction of 24-hour urinary total protein to <3.5g in patients with baseline nephrotic range proteinuria (urinary total protein ≥ 3.5g) or decrease in urinary total protein by >50% in patients with sub-nephrotic proteinuria (urinary total protein <3.5g) or stabilization (+/- 25%) or reduction of serum creatinine and rise of eGFR from the baseline value.	Primary end point: End of 3 month–CYC 47.6% vs. MMF 33.3%, p =0.454 End of 6 month–CYC 19% VS. 28.6%, *p =* 0.572 Secondary end point: End of 3 month – CYC 28.6% vs. 47.6% (no p value) End of 6 month - 66.7% in each arm (p value not stated) Non-responders End of 3 months – CYC 23.5% vs. MMF 19% (p value not stated) End of 6 months - CYC 14.3% vs. MMF 4.8% (*p* value not stated)	Significant adverse events: Alopecia CYC 76.2% vs. MMF 0% p value <0.001 Nausea/vomiting CYC 76.2% vs. MMF 0% *p* < 0.001 Infection related adverse effects were comparable in both groups (10 in CYC vs. 7 in MMF). Urinary tract infection–CYC 19.04% vs. MMF 9.52% *p* value 0.796 OR 0.473 95% CI 0.083–3.492 Herpes Zoster–CYC 14.3% vs. CYC 14.3% p value 0.337 OR 1 95% CI 0.178–5.632 Chest infection–CYC 14.3% vs. MMF 9.5% p value 0.328 OR 0.632 95% CI 0.094–4.230	1b
			Secondary end point: Return of serum creatinine to previous baseline, plus a decline in the 24-h urinary total protein <500 mg All end points were assessed at 3 and 6 months.			
Comparison of low-dose intravenous cyclophosphamide with oral mycophenolate mofetil in the treatment of lupus nephritis. Rathi et al. (42) Study type: Randomized trial Type of LN:Class III, IV or V	Adult study including some JSLE patients Total: 100 F 92 M 5 Age: years +/- SD Cyclophosphamide 30.6 +/- 9.5 MMF 28.3 +/- 9.5	*Induction:* MM−500 mg twice daily and increased every 2 weeks to achieve a target dose of 1.5–3.0 g/day depending on leukocyte count/tolerability. At 2 weeks, the dose was increased to 1,000 mg twice daily; increased to 1,500 mg twice daily at 4 weeks. In case of GI intolerance, the frequency was changed to three time daily, followed by change of formulation if required. In case of persistence symptoms dose was reduced stepwise by 25%. or CYC – six fortnightly at a fixed dose of 500mg each Other drugs: Steroids Hydroxychloroquine ACE or ARB At end of induction (6m in MMF group; 3m in CYC group), all patients started maintenance azathioprine	Primary outcome: ‘treatment response’ defined as a decrease in the urinary PCR to <3 in subjects with a baseline ratio ≥3 or a decrease in urinary PCR by ≥50% in those with a baseline ratio <3, along with stabilization or improvement in serum creatinine (a 24-week serum creatinine level within 25% of baseline). Secondary outcomes: Complete renal remission – defined as return to normal serum creatinine along with proteinuria <0.5g/day and inactive urine sediment SELENA–SLEDAI score - Adverse events	At 24 weeks, 37/50 (74%) patients in each group achieved the primary end point (OR 1.0 95% CI 0.37–2.70 *p =* 1.0). ITT analysis. Complete renal remission rate: 25/50 (50%) in CYC vs. 27/50 (54%) in MMF group OR 1.17 95% CI 0.50–2.77 p value 0.84. ITT analysis.	Gastrointestinal symptoms–significantly more frequent in patients receiving MMF (52 vs. 4%, p <0.001). Other adverse events were similar.	1b
Mycophenolate Mofetil or Cyclophosphamide in Indian Patients with Lupus Nephritis: Which is better? A Single-Center Experience. Mendonca et al. (43) Study type: prospective randomized open label trial Type of LN: Biopsy Proven. Class III/IV.	Adult study including some JSLE patients Total: 40 F 32 M 8 Age (year) MMF group: 26.0 +/- 10.8 IVC: 25.7+/- 10.3	MMF (titrated from 750 mg twice daily in 1st week and 1 g twice daily in 2nd week to a target dose of 1.5 g twice daily if required based on disease activity and response. Reduction was permitted to 2g/day in response to adverse events) Or CYC 750 mg/m2 of body surface area, adjusted to 500–1,000 mg/m2 of body surface area every 4 weeks to maintain a nadir leukocyte count of 2.5–4.0 x 10 9/L for a total of 6 pulses. A 25% decrease in dose for patients >60 years and serum creatinine >3.4 mg/dL. Other drugs: Steroids ACE/ARB Statin/alternative for hyperlipidaemia	Primary outcome: Response to therapy at 6 months–not clearly defined Secondary outcomes: Complete remission, SLEDAI, adverse events	Response to therapy–MMF 88.24% and CYC 86.95% - not clear what was measured Complete remission after 6 months: MMF 9/17 (52.94%) and CYC 11/23 (47.82%) *p =* 0.861 Partial remission after 6 months: MMF 6/17 (35.3%) and CYC 9/23 (39.13%) *p =* 0.861 Patients failing to achieve complete remission at 6 months: MMF 8/8 (100%) and CYC 12/12 (100%) *p =* 1.000 SLEDAI – MMF 4.1 and CYC 3.8 *p =* 0.14	Adverse events were comparable in both groups with vomiting being more common in the CYP group (CYC 10/23 (43%) and MMF 2/17 (12%) *p =* 0.041) whereas diarrhea was more frequent in the MMF group (MMF 5/17 (29%) and 3/23 (13%) *p =* 0.249) The rate of opportunistic infections was comparable between the groups: Urinary tract infections MMF 1/17 (6%) vs. CYC 2/23 (9%) *p =* < 0.0001 Herpes zoster MMF 2/17 (12%) vs. CYC 3/23 (13%) *p =* 1.000 Tuberculosis MMF 0/17 (0%) vs. CYC 1/23 (4%) *p =* 0.421	2B
Efficacy of mycophenolate mofetil in adolescent patients with lupus nephritis: evidence from a two-phase, prospective randomized trial. Sundel et al. (44) Study type*:* Prospective randomized trial Type of LN: active type III-V	Adolescent study Total: 40 Induction phase <18 year old 24 Maintenance <18 years old 16 Mean age (years) Induction: MMF 14.9 IV CYC: 14.6 Maintenance: MMF 13.9 AZA 15	*Induction* MMF (target dose 3.0g/day) or IV CYC (0.5-1.0 g/m2/month) plus prednisolone *Maintenance* Oral MMF (1g twice daily) or oral AZA (2 mg/kg/day) plus prednisolone.	Treatment response - decrease in urine PCR measured over 24 h to <3 in patients with baseline nephrotic range proteinuria urine PCR ≥3, or by ≥50% in patients with sub-nephrotic baseline urine PCR ≤ 3, and stabilization (+/-25%) or improvement in serum creatinine. Maintenance: The time to treatment failure, measured as time to death, ESRD, sustained doubling of serum creatinine, renal flare (proteinuric or nephritic), requirement for rescue treatment (corticosteroid, plasmapheresis, intravenous immunoglobulin, or non-protocol immunosuppressants) to manage exacerbation, or deterioration of LN.	Induction: 15/24 (62.5%) achieved the treatment response. In the MMF group 7/10 (70%) were classed as responders at 6 months compared with the CYC group 8/14 (57.1%) (OR 2.0 95% CI 0.2-15.5 *p =* 0.53). Maintenance: Fewer patients treated with MMF experienced treatment failure [1/8 (12.5%)] compared with those treated with azathioprine [5/8 (62.5%)]. No p-value stated.	During both phases, rates of serious adverse events were similar in both study arms.	2B
Comparing the efficacy of low-dose vs. high-dose cyclophosphamide regimen as induction therapy in the treatment of proliferative lupus nephritis: a single center study. Mehra et al. (45) Study type: Investigator-initiated, open label, parallel group randomized controlled trial Type of LN: biopsy proven proliferative lupus glomerulonephritis of class III, IV according to ISN/RPS	Adult study including some JSLE patients Total 75 F 68 M 7 Age of SLE onset, Yrs Mean +/- SD Low dose 28.5 +/- 10.05 High dose 25.7 +/- 10.35 Age of onset of LN, Yrs Mean +/- SD Low dose 30.71 +/- 10.04 High dose 27.24 +/- 10.60	*Induction* Low dose CYC or high-dose CYC regimen Other drugs include: Steroids Azathioprine Hydroxychloroquine Co-trimoxazole prophylaxis For hypertension, ACE/another appropriate drugs For hyperlipidaemia, atorvastatin In patients who did not respond to CYC, MMF rescue therapy was instigated, and the patient was discontinued from study.	Complete renal response Partial renal response No renal response Secondary outcomes: Patients with renal and non-renal disease flares	At 52 weeks: 27/75 (73%) in high dose group achieved complete/partial renal response vs. 19 (50%) in low dose (*p* = 0.04). The proportion of patients achieving a complete renal response was comparable between the treatment arms [24 (65%) vs. 17 (44%), *p =* 0.08]. The proportion of non-responders in the high dose group was significantly lower [10 (27%) vs. low dose 19 (50%) (*p* = 0.04)]. Renal relapses were higher in the low dose group vs. high dose [9 (24%) vs. 1(3%), (*p* = 0.01)].	There was significant alopecia and CYC-induced leucopenia in the high dose group	1B
Outcome of low dose cyclophosphamide for induction phase treatment of lupus nephritis, a single center study Sigdel et al. (46) Study type: prospective observational study Type of LN: biopsy-proven nephritis (class III, IV, V or mixed)	Adult study including some JSLE patients Total: 41 F:M ratio 12.6:1 Mean age 29.6 +/- 10.6 years (range 14–61 years) 22 patients (53.7%) had class IV nephritis.	*Induction* IV Cyclophosphamide 500 mg monthly for 6 months. Other drugs: Steroids Hydroxychloroquine Calcium PPI ACE/ARB prophylactic co- trimoxazole	Complete renal remission Partial renal remission Renal response–defined as either Complete or Partial remission.	18/41 patients (43.9%) achieved complete remission, 16/41 (39.0%) achieved partial remission, yielding an overall renal response rate of 82.9%. Nephrotic range proteinuria (Urinary total protein ≥3 g/day) and severe hypoalbuminemia (serum albumin <20 g/L) at baseline influenced achievement of complete renal remission (*p <* 0.05).	Infection - seen in 12 patients (29.3%). Four deaths (9.6%) observed, all due to infection.	3
Renal Outcome in Patients with Lupus Nephritis Using a Steroid-free Regimen of Monthly Intravenous Cyclophosphamide: A Prospective Observational Study	Adult study including some JSLE patients	*Induction for patients with first episode of LN (<6 months):* IV CYC and low dose glucocorticoids. Glucocorticoid dose based on extrarenal manifestations.	Complete renal response	Complete remission - achieved in 25/40 (62.5%) and partial remission in 8/40 (20%)	21/40 (52.5%) developed at least 1 infection.	2B
Fischer-Betz et al. (47) Study type: prospective observational study Type of LN: WHO class III, IV, V First episode of LN (duration <6 months); excluded relapses	Total: 40 F 33 M 7 Age, yrs 29.7 +/- 10.1	Other drugs: All patients received mesna For hypertension–ACE/ARB	Partial renal response Non responder	Mean starting dose of prednisone was 23.9+/23.8 mg/day. In a posthoc analysis the authors separately analyzed patients initially treated with prednisone doses ≥20 mg/day (Group A, n = 19) or <20 mg/day (Group B, n = 21). Complete renal response was achieved in 52.6% (Group A) vs. 71.4% (Group B; p = 0.37); and PR in 26.3% vs. 14.3%, respectively (p = 0.58).	No significant differences concerning the rate of infections in relation to the initial prednisone dose [61.4% (high-dose) and 52% (low-dose)].	

*LN, lupus nephritis; M, Male; F, female; CYC, cyclophosphamide; AZA, azathioprine; MMF, mycophenolate mofetil; PCR, protein creatinine ratio; PPI, protein pump inhibitor; ACE, angiotensin converting enzyme; ARB, angiotensin receptor blocker; IV, intravenous; ISN, International Society of Nephrology; RPS, Renal Pathology Society; SLEDAI score, Systemic Lupus Erythematosus Disease Activity Index scores; SELENA, Safety of Exogenous Estrogens in Lupus Erythematosus National Assessment; ITT, intention to treat analysis; BILAG, British Isle Lupus Assessment; SLICC SDI, Systemic Lupus International Collaborating Clinics Damage Index*.

### MMF vs. Cyclophosphamide Treatment in LN

*Pediatric evidence*–The largest exclusively pediatric study is from the UK JSLE Cohort Study, comparing MMF and cyclophosphamide as induction treatments for proliferative LN. 34/51 (67%) of patients received MMF and 17/51 (33%) received cyclophosphamide (56). No significant differences were identified between the treatment groups in terms of their numerical BILAG disease activity scores, urine albumin/creatinine ratio, serum creatinine, ESR, anti-dsDNA antibody, C3 levels and patient/physician global scores at 4–8 months, 10–14 months after renal biopsy, and at last follow up. There were also no differences in SLICC-SDI scores at 13-months, and last follow up. The time to achievement of inactive LN, and time to subsequent renal flare was also comparable between the groups (Table 2) (56). Despite being the largest JSLE study comparing MMF/cyclophosphamide LN induction treatment to date, this study was limited by its numbers, particularly in comparison to adult SLE studies.

*Mixed pediatric and adult SLE evidence*–In a study including 35 Nepalese patients (mean age 25.43 ± 10.17–years), Sedhain et al. demonstrated low dose MMF (maximum daily dose of 1.5 g) to be comparable to monthly cyclophosphamide pulses (dose of 0.5–1g/m^2^) as induction treatment for proliferative LN (41). Both treatments led to similar reductions in proteinuria, improvements in kidney function (serum creatinine, eGFR) and achievement of complete remission, with less adverse events in the MMF group. Rathi et al. randomized 100 SLE patients (mean age 28.3-years) to low dose cyclophosphamide (six fortnightly infusions of 500 mg each) or MMF (daily doses 1.5–3g), accompanied by three intravenous methylprednisolone infusions, followed by oral corticosteroids. Maintenance therapy of azathioprine and low-dose corticosteroid was started after 6-months of induction therapy. They demonstrated similar rates of treatment response in each group (*p* = 1.0), with complete renal remission achieved by 50% of the cyclophosphamide group, and 54% of the MMF group (*p* = 0.84) after 24-weeks treatment. They concluded that low dose cyclophosphamide is comparable in safety and efficacy to oral MMF as an induction treatment for less severe LN (class III, IV, V LN, but excluding those with crescentic LN or a serum creatinine over 265 μmol/l) (42). Mendonca et al. conducted a 24-week prospective, randomized, open-label trial comparing oral MMF with monthly IV cyclophosphamide as induction therapy for active biopsy proven Class III and IV LN, in 40 Indian patients. MMF and cyclophosphamide were demonstrated to be comparable in terms of the rates of complete remission, partial remission and cumulative probability of response at 6 months (Table 2) (43).

The Aspreva Lupus Management Study (ALMS trial) published it's result in late 2011 (57). ALMS was a large, multinational, prospective, two-stage, parallel-group, phase III RCT including patients with LN>12-years old. In the first phase of the study (24-week induction), patients were randomized to oral MMF (target dose 3 g/day) or intravenous cyclophosphamide (0.5–1 g/m^2^/month), plus prednisone. Responders then went into phase 2 of the study (36-month maintenance), where patients were randomized 1:1 to MMF (1.0 g, bd) or oral azathioprine (AZA) (2 mg/kg/day), plus prednisone. Of the 370 patients enrolled, 24 were aged <18-years (mean age of 14.8, standard deviation 1.48-years), and the results of sub-analyses for this age group were published in 2012, showing induction treatment with MMF and intravenous cyclophosphamide to be equally efficacious. During the maintenance phase, MMF was demonstrated to be at least as effective as azathioprine. The results of the JSLE patients were largely comparable to those of the adult SLE patients, but of note, adolescent patients more commonly developed serious infections, regardless of the treatment arm (Table 2) (44). Whilst the results of this study are encouraging, larger trials involving purely pediatric and adolescent study populations are needed.

### Cyclophosphamide Treatment for LN

A single center RCT has compared high dose cyclophosphamide (six four-weekly cycles of 750 mg/m^2^, maximum of 1.5 g/pulse) with low dose cyclophosphamide (six fortnightly cycles of 500mg). This study included 75 proliferative LN patients (mean age 30.7±10.04-years, standard deviation years in the low dose cyclophosphamide group, and 27.24±10.60-years in the high dose group). At 52-weeks, high dose cyclophosphamide was shown to be more effective than low dose in achieving a partial and complete response (73 vs. 50%, *p* = 0.04), and in preventing LN relapse (3 vs. 24%, *p* = 0.01). There was a significantly lower number of non-responders in the high dose cyclophosphamide group (27 vs. 50%, *p* = 0.04, Table 2) (45). Further studies involving multiple centers and younger patients are required, as this was single center study involving both JSLE and adult SLE patients.

A JSLE and adult-SLE Nepalese prospective observational study has assessed the performance of an unconventional cyclophosphamide regimen which differs to the more commonly used Euro Lupus (500 mg every 2-weeks for 3-months) (58) or the National Institute of Health (NIH) regimens (0.5–1g/m^2^ monthly for 6-months) (59). In the Nepalese study 500 mg of cyclophosphamide was given per month, for 6-months. The study included 41 patients with a mean age of 26.9 ± 10.6-years, with biopsy proven class III, IV, V, or mixed III/IV+V LN. 43.9% of patients achieved complete remission and 39% achieved partial remission (overall response rate of 83%) using this cyclophosphamide regimen (Table 2) (46). The overall response rate of 82.9% is comparable to those of the Euro Lupus trial where 71% of the low-dose cyclophosphamide group achieved renal remission (58) and the NIH trial where 85% achieved renal remission (60).

Intravenous cyclophosphamide is usually combined with high dose intravenous methylprednisolone or oral corticosteroids for the management of LN. A prospective observational study evaluated the use of IV cyclophosphamide without additional methylprednisolone/high dose oral prednisolone in patients presenting with their first episode of LN. In this study, the use and dose of prednisone was based solely on the presence of mild to moderate extrarenal SLE manifestations, with dose tapering decided upon based upon extrarenal activity alone. Fourty patients with a mean age of 29.7 ± 10.1-years received 12 IV cyclophosphamide pulses over 24-months (6-monthly pulses, and six quarterly pulses). The initial cyclophosphamide dose was 0.5 g/m^2^; subsequent doses were increased by 250 mg, with a maximum of 1,500 mg per pulse. After 24-months, 62.5% of patients met the criteria for complete renal response and 20% met the criteria for partial renal response. Mean starting dose of prednisone was 23.9+/-23.8 mg/day. *Post-hoc* analysis compared outcomes for patients treated with prednisone doses ≥20 mg/day (Group A, *n* = 19) and <20 mg/day (Group B, *n* = 21). Complete renal response was achieved in 52.6% of Group A patients vs. 71.4% of Group B patients (*p* = 0.37); and partial renal response was seen in 26.3 vs. 14.3% of group A and B patients respectively (*p* = 0.58). Overall, renal outcomes were the same irrespective of initial prednisone doses (*p* = 0.46, Table 2) (47). These findings warrant further exploration in JSLE, ideally within a randomized trial comparing different corticosteroid dosing regimens in children and young people with LN.

### Rituximab

A pediatric study including 44 JSLE patients with active LN (ISN RPS class III/IV/V) aged 3.5–13.8-years (median 8.4) compared outcomes in patients treated with induction treatment consisting of methylprednisolone followed by either rituximab (*n* = 17), MMF (*n* = 12) or IV cyclophosphamide (*n* = 15), with a tapering dose of oral prednisolone. MMF was added as maintenance immunosuppression (800 mg/m^2^ daily) in all children from 3-months. At 36-months, flare-free survival was highest in the rituximab group than other treatment groups (100% RTX vs. 83% MMF vs. 53% for CYC, *p* = 0·006). The mean daily dose of prednisolone was also significantly lower in the rituximab group after 3-months (rituximab vs. MMF, *p* = 0.005; rituximab vs. cyclophosphamide, *p* = 0.0001). There was a numerical difference in the proportion of patients achieving complete remission (76.5% achieved complete remission with rituximab, 41.7% with MMF and 46.7% with cyclophosphamide, however this did not reach statistical significance (*p* = 0.28, Table 3) (48).

**Table 3:** Summary of pediatric lupus nephritis treatment studies from 2012 including biologics, disease modifying anti-rheumatic drugs and mesenchymal stem cell therapy.

**Study**	**Patients**	**treatment**	**Outcome measures**	**Main result(s)**	**Safety outcomes**	**Level of evidence**
Efficacy and safety of rituximab in comparison with common induction therapies in pediatric active lupus nephritis. Basu et al. (47) Study type*:* Retrospective cohort study Type of LN: active LN - class IIIA or IIIA/C (±V); class IVA or IVA/C (±V) LN, and pure class V nephritis with nephrotic- range proteinuria (ISN/RPS classification)	Total: 32 Pediatric study Rituximab 17 MMF 12 CYC 15 Age (year); mean (SD) Rituximab: 8.4 (4.6) MMF: 8.1 (3.2) CYC: 8.7 (4.1)	Induction therapy: Methylprednisolone pulses (15 mg/kg daily for 3 days) followed by either two rituximab pulses (375 mg/m^2^ weekly) or MMF (1,200 mg/mt^2^ daily) or six pulses of CYC (500 mg/m^2^ once very fortnight) with prednisolone 2 mg/kg daily for 1 month and then weaned at the discretion of the clinicians. MMF was added as maintenance (800 mg/m^2^ daily) in all children from the third month onwards	Primary outcome: flare-free survival LN flare defined if there was reappearance or deterioration of clinical manifestations of LN and renal biochemical parameters (≥25% decrease in baseline eGFR or proteinuria ≥1g/24h) along with rising titres of immunological parameters after initial postinduction stabilization or improvement. Secondary outcomes: Overall patient survival, renal survival, time to first flare after induction, number of flares, drug-related adverse reactions	Flare-free survival was significantly higher at 36 months with rituximab compared with MMF and CYC (100% for rituximab vs. 83% for MMF and 53% for CYC, *p* = 0.006). 13/17 (76.5%) achieved complete remission with rituximab compared with 5/12 (41.7%) and 7/15 (46.7%) with MMF and CYC, respectively, at last follow-up. Mean daily prednisone dosage was significantly lower in rituximab treated patients [rituximab vs. MMF, p = 0.005, Rituximab vs. CYC, p = 0.0001] at 36 months.	Adverse events were reported in 5/17 (29.4%) in the rituximab group compared with 7/12 (58.3%) in MMF and 15/15 (100%) in CYC group (no p values stated) No serious adverse events occurred after rituximab or MMF therapy.	3
Efficacy and Safety of Rituximab in Patients With Active Proliferative Lupus Nephritis. The Lupus Nephritis Assessment With Rituximab Study Rovin et al. (49) Study type: randomized, double-blind, placebo controlled phase III trial Type of LN: class III or IV LN	Adult study including some JSLE patients Total: 144 F 130 M 14 Age, mean +/- SD Placebo: 29.4 +/- 9.3 Rituximab: 31.8 +/- 9.6	*Induction* Placebo or rituximab 1,000mg administered intravenously on days 1, 15, 168, and 182. MMF was initiated at 1.5 gm/day in 3 divided doses and the dosage increased to 3 gm/day by week 4 as tolerated. This was continued to at least week 52. Methylprednisolone 1,000 mg was administered IV 30–60 min prior to study drug on day 1 and again within 3 days as therapy for active LN. To prevent infusion reactions, methylprednisolone 100 mg was given intravenously 30–60 min prior to the administration of study drug on day 15, 168, 182.	Primary end point: Complete renal response Partial renal response No renal response Secondary end points: Clinical:Number of patients with a baseline urinary PCR of >3 who achieved a UPC ratio of <1 at week 52 Median number of months to first complete response ratio Time adjusted AUCMB of BILAG index global score Change from baseline to week 52 in the SF-36 physical function score Achievement of a complete renal response from week 24 to week 52 Achievement of complete renal response at week 52 Serological Relative change from baseline in anti-dsDNA Change in baseline C3 and C4	Primary end point: Renal response rates (complete, partial and no response rate) at week 52 showed no statically significant difference between rituximab and placebo groups (*p =* 0.55) The overall (complete and partial) renal response rate was 45.8% for placebo and 56.9% for rituximab treated patients (*P* = 0.18). Partial renal responses accounted for most of the difference. Secondary end points: Clinical: No statistically significant difference between rituximab and placebo group Serological: Statistically significant improvements in C3, C4 and ds-DNA levels were observed amongst patients treated with rituximab. Eight placebo-treated patients and no rituximab-treated patients required cyclophosphamide rescue therapy.	The rates of serious adverse events, including infections, were similar in both groups. Neutropenia, leukopenia, and hypotension occurred more frequently in the rituximab group.	1b
		Oral prednisolone 0.75 mg/kg/day (maximum 60mg) was administered until day 16 and tapered to ≤ 10 mg/day by week 16. Other immunosuppressants in addition to steroids and MMF were not permitted and discontinued during the screening period. If a new immunosuppressant agent and/or high dose steroids for >2 weeks were used, the study subject was classed as a non-responder. ACE/ARBs had to initiated at least 10 days before randomization. Antimalarials had to be maintained at a constant dose if used. NSAIDs were prohibited.				
Efficacy and safety of rituximab in Japanese patients with systemic lupus erythematosus and lupus nephritis who are refractory to conventional therapy Tanaka et al. (50) Study type: multicentre, open label, phase II clinical trial Type of LN: any	Adult study including some JSLE patients Total: 34 No details on gender No details regarding age – inclusion criteria: patients aged 16-75 years	Rituximab - 1,000 mg given 2 weeks apart (days 1 and 15), repeated after 6 months (days 169 and 183) Before each rituximab infusion: acetaminophen, chlorpheniramine maleate and methylprednisolone Other drugs: Corticosteroid and any concomitant immunosuppressant at a stable dose before study entry.	Renal responses: complete, partial or no renal response based on LUNAR (Lupus nephritis assessment with rituximab) [1] and ACR (American College of Rheumatology) guidelines. [2] Overall renal response = complete and partial Change in BILAG scores Disease remission: change in BILAG A or B score to a BILAG C or D score in every organ system. Partial remission: change in BILAG A or B score to a C or D score in least one organ system but with presence of one BILAG A or B score in another organ system. No improvement: BILAG A or B score that remained unchanged at week 53.	Renal responses:In 17 patients with LN, overall renal response rates of 58.8% (95% CI 32.9–81.6) and 52.9% (95% CI 27.8 −77) by ACR and LUNAR criteria respectively were seen. The median value of urinary PCR/urinary creatinine ratio decreased from 2.2 (IQR 1.4-3.8) at baseline to 0.4 (IQR 0.10-2.44) at week 53 *p =* 0.0068). eGFR remained stable with a median value of 71.3mL/min/1.73m2 (IQR 41.2 – 101.5) at baseline vs. 72.3 mL/min/1.73m2 (IQR 56.8-93.0) at week 53 *p =* 0.1928).	Rituximab was well tolerated, and most adverse drug reactions were grade 1 – 2 in severity.	2B
			For patients with involvement of one study organ, remission was a change from a BILAG A or B score to C or D score and partial remission was a change from a BILAG A score to B score.	BILAG scores: 24/34 (76.5%) responded to rituximab therapy at week 53; 16/34 (47.1%) achieved remission and 10/34 (29.4%) achieved partial remission. BILAG global score in 34 patients decreased significantly from a median of 12.5 (interquartile range (IQR) 10-14) at baseline to 3.5 (IQR 1-6) at week 53 (p <0.0001). A significant reduction in concomitant prednisolone was achieved – 45mg/kg/day (IQR 35-55) at baseline to 6mg/kg/day (IQR 5 – 8.9) at week 53 (p <0.0001). Serological improvements were seen: C3 levels (69mg/dL (IQR: 48.8 – 82.0) at baseline vs. 88.5mg/dL (IQR 81.5 – 103.8) at week 53 p <0.0001) C4 levels (16.5mg/dL (IQR 8 – 332) at baseline vs. 22mg/dL (IQR 18-28) at week 53 *p <* 0.0001, data not shown) CH50 (31.2/mL (IQR 14.7-39.4) at baseline vs. 39.0/mL (IQR 34 – 46.7) at week 53 *p =* 0.0027, data not shown) Anti-dsDNA–(20.5 IU/mL (IQR 10 – 67.8) at baseline vs. 10 IU/ml (IQR 10-12.8) at week 53 *p <* 0.0001)		
Efficacy and Safety of Ocrelizumab in Active Proliferative Lupus Nephritis Mysler et al. (51)	Adult study including some JSLE patients Total 381 M 49 F 332	In patients with active proliferative LN, placebo or 400mg ocrelizumab or 1,000mg ocrelizumab given as an infusion on days 1 and 15, followed by an infusion at week 16 and every 16 weeks thereafter.	At week 48: Complete renal response Partial renal response Nonresponse	Overall renal response: 54.7% - placebo-treated 66.7%−400 mg ocrelizumab-treated 67.1%−1,000 mg ocrelizumab-treated 66.9%–combined ocrelizumab- treated groups.	Serious adverse events: 27.2% placebo-treated patients 35.7% 400 mg ocrelizumab–treated patients, 22.0% 1,000 mg ocrelizumab–treated patients.	1b
Study type*:* Randomized, double-blind phase III study Type of LN: active proliferative	Age mean (range) years 31.3 (16–69) <30 years, % 50	Patients also received ELNT regimen induction treatment (i.e., CYC 500mg IV every 2 weeks for 6 months) or MMF as induction therapy	There was trend (*p =* 0.065) toward greater overall renal response rates at 48 weeks with ocrelizumab treatment and the ELNT regimen vs. placebo treatment and the ELNT regimen. Add ocrelizumab to background MMF had little effect on the overall renal response with adjusted treatment differences (vs. MMF alone) of −0.3% (95% CI −20.0, 19.7) and 13.3% (95% CI −6.0, 32.6) for the 400 mg ocrelizumab-treated and 1,000 mg ocrelizumab-treated groups respectively.	Serious infection rates (events/100 patient-years): 18.7 (95% CI 12.2, 28.7) placebo-treated patients 28.8 (95% CI 20.6, 40.3) 400 mg ocrelizumab-treated patients 25.1 (95% CI 17.4, 36.1) 1,000 mg ocrelizumab-treated patients. Patients receiving background MMF who received ocrelizumab had high serious infection rates per 100 patient-years (34.5 [95% CI 23.5, 50.7] and 28.6 [95% CI 18.6, 43.8] for 400 mg ocrelizumab and 1,000 mg ocrelizumab respectively) than those who received placebo (19.4 [95% CI 11.5, 32.7). Study terminated early due to higher rate of serious infections.	

*LN, lupus nephritis; M, Male; F, female; MMF, mycophenolate mofetil; PCR, protein creatinine ratio; ESRD, End stage renal disease; BILAG, British Isle Lupus Assessment; ACE, angiotensin converting enzyme; ARB, angiotensin receptor blocker; CYC, cyclophosphamide; ELNT, Euro Lupus Nephritis treatment regimen; BILAG, British Isle Lupus Assessment; NSAID, nonsteroidal anti-inflammatory drugs; TAC, tacrolimus; AZA, azathioprine; ECLAM, European Consensus Lupus Activity Measurement; SLEDAI score, Systemic Lupus Erythematosus Disease Activity Index scores; UPC ratio, urine protein creatinine ratio*.

In a study including 144 JSLE and adult SLE patients (mean age 30.6-years), Rovin et al. evaluated the efficacy and safety of rituximab in a randomized, double-blind, placebo-controlled phase III trial in patients with class III and IV LN treated concomitantly with MMF and corticosteroids. Patients received rituximab (1,000 mg) or placebo on days 1, 15, 168, and 182. The primary end point of the study was assessment of renal response status at 12-months. The overall (complete and partial) renal response was 56.9% in the patients treated with rituximab (in addition to a background of MMF and corticosteroids) and 45.8% in the patients receiving placebo (*p* = 0.18). Of note, more placebo treated patients required cyclophosphamide rescue therapy during the 12-months of follow-up, and there were significantly greater reductions in anti-dsDNA and C3/C4 levels in patients receiving rituximab. The study also showed that combination of rituximab with MMF and corticosteroids did not result in any new or unexpected safety alerts (Table 3) (49).

In 2016, Tanaka et al. investigated the efficacy and safety of rituximab in an open-label study of 34 Japanese patients with active SLE (17/34 with LN) who had been refractory to conventional therapy. The study included JSLE patients who were >16-years, but did not specify how many were recruited or the mean age of the study population. 76.5% of these previously refractory patients responded to rituximab therapy at week 53; with 47.1% achieving remission (defined as a change from British Isles Lupus Assessment Grade (BILAG) A or B score to a BILAG C or D score in every organ system) and 29.4% achieving partial remission (change from a BILAG A or B score to a C or D score in at least one organ system, but with presence of one BILAG A or B score in another organ system). In the patients with LN, 52.9% of patients demonstrated an overall renal response (29.4% complete renal response, 23.5% partial renal response) at 52-weeks. The response rate was higher in patients with biopsy proven class III/IV LN than other LN patients. A significant reduction in prednisolone was observed following rituximab treatment (45 mg/day, inter-quartile range, IQR: 35–55) at baseline to 6 mg/day (IQR: 5–9) at week-53. Most adverse events were graded mild to moderate, however there were a few serious adverse events (cerebral infarction, cholecystitis, endometritis, and hypoferric anemia) which were likely associated with the underlying diseases/concomitant illnesses rather than rituximab (Table 3) (50).

### Ocrelizumab

Ocrelizumab is a recombinant humanized monoclonal antibody that selectively targets and depletes CD20+ B-cells in the peripheral circulation. A randomized, double blind phase III study has compared patients treated with placebo or IV ocrelizumab (either 400 or 1,000 mg) in addition to standard care, which comprised of corticosteroids plus either MMF or Euro-Lupus regimen treatment (cyclophosphamide induction and azathioprine maintenance treatment). The overall renal response rates were not significantly different between treatment groups, and ocrelizumab was associated with a higher rate of serious infections leading the study to be terminated early (Table 3) (51).

### Tacrolimus

Calcineurin inhibitors such as cyclosporin A and tacrolimus have been investigated in several studies, both in isolation and as part of a multitarget regimen in adult SLE (61–64). In 2013 Tanaka et al. published a small open-label, prospective, long-term tacrolimus-based treatment study involving 19 young patients (mean age 18-years) with biopsy proven LN. 15/19 (79%) had a history of LN and experienced a “lupus flare”, defined as a sustained increase in urinary protein excretion by more than 25% of the baseline value, associated with a significant decrease in serum C3 levels and/or increase in the serum anti-dsDNA antibody titer, and/or other signs of active SLE. Their usual cytotoxic was discontinued and replaced by tacrolimus (3 mg/day) with concomitant prednisolone (maximum 30 mg). 4/19 patients had new onset LN and were treated with a multitarget regimen consisting of Tacrolimus plus mizoribine (selective inhibitor of inosine monophosphate dehydrogenase in the purine synthesis pathway, acting in a similar manner to MMF) in combination with prednisolone. 12/19 (63%) achieved a complete renal response and 5/19 (26%) demonstrated a partial response, with two patients showing no response. There were no serious adverse effects (Table 4) (52). This study is of interest as Tacrolimus use is not reported frequently in JSLE, however, it is clearly limited by the sample size, lacks a wash out period and blinding.

**Table 4:** Summary of pediatric lupus nephritis treatment studies from 2012 including tacrolimus or mesenchymal stem cell therapy.

**Study**	**Patients**	**Treatment**	**Outcome measures**	**Main result(s)**	**Safety outcomes**	**Level of evidence**
Long-Term Tacrolimus-Based Immunosuppressive Treatment for Young People with Lupus Nephritis: A Prospective Study in Daily Practice. Tanaka et al. (52) Study type*:* Open label prospective study Type of LN: any proven-on biopsy	Adult study including some JSLE patients Total 19 M 6 F 13 Median age: 18 (range 9–38 years)	*Re- induction and maintenance* When a lupus flare was diagnosed, the previous cytotoxic agent was replaced by TAC. Other drugs: Prednisolone and MZR depending on clinical picture	Complete, partial or no renal response. Urinary PCR, serum C3 level, serum CH50 value, anti-dsDNA antibody, serum creatinine, ECLAM index (SLE disease activity), prednisolone dose–Measured at baseline, 3, 6, 12, 24 and 36 months and last visit.	Complete response−12/19 (63%) Partial response−5/19 (26%) No response−2/19 (11%) Despite tapering of prednisolone, a marked improvement compared with baseline values was observed in all laboratory results as early as 3 months after the initiation of TAC. Sustained improvements in the outcome measures compared with baseline values and after a mean of 42 months of treatment: ECLAM index, serum CH50, anti-dsDNA antibody (all *p <* 0.01), urinary PCR, serum C3 level (both p <0.05). Serum creatinine level remained within the normal range in all patients.	No serious adverse effects were observed.	2B
Outcomes of maintenance therapy with tacrolimus vs. azathioprine for active lupus nephritis: a multicenter randomized clinical trial. Chen et al. (53) Study type*:* Prospective randomized, open label and controlled trial Type of LN: active LN (ISN/RPS Classes III, IV or V)	Adult study including some JSLE patients Total 70 F 61 M 9 Age, mean +/- SD Tac 30.7 +/- 10.2 Aza 33.1 +/- 10.9	*Maintenance:* TAC plus prednisone (TAC group) or AZA plus prednisone (AZA group). Other drugs: ACE/ARB Statins/fibric acid derivatives	Primary outcome: incidence of renal relapse Secondary outcome: ‘maintaining’ response (defined as complete or partial remission), changes of clinical parameters (including proteinuria, serum albumin, serum creatinine, eGFR and serum C3), and adverse effects (including leucopenia, infections, gastrointestinal complaints, liver function disorder and nephrotoxicity)	After six months of therapy, two patients in AZA group developed renal relapse compared to none of the TAC group [*p =* 0.49; odds ratio, 1.06; 95% CI (0.98, 1.15)].	Leucopenia was significantly more frequent in the AZA group than the TAC group (47% vs. 9%, *p <* 0.001).	1B
Low-dose tacrolimus in treating lupus nephritis refractory to cyclophosphamide: a prospective cohort study. Fei et al. (54) Study type*:* Prospective cohort study Type of LN: Refractory LN (Class III, IV, V, III+V, IV+V, unknown) resistant to CYC *Refractory (*failed CYC >8 g over 6 months induction treatment)	Total: 26 Adult study including some JSLE patients F 22 M 4 Age 29.36 +/- 9.45 years	TAC (initial dose of 2 mg/day (body weight <60 kg) or 3 mg/day [body weight ≥60 mg)] and prednisolone Other drugs: Pre-existing drugs such as ACE/ARBs were maintained throughout	Primary end point: Complete remission following 6 months of treatment. Secondary end points: complete or partial remission, changes in serum creatinine, serum C3 values, 24-h urinary protein excretion, and adverse effects	Complete remission at 6 months: 10/26 patients (38.5%) Partial remission at 6 months: 13/26 (50%) Mean urinary protein significantly decreased from 6.91 ± 4.50 g at baseline to 1.11 ± 1.10 g at 6 months (*p <* 0.001). Mean serum albumin level significantly increased from 25.56 ± 7.94 g/L at baseline to 38.12 ± 2.42 g/L at 6 months (*p <* 0.001). Mean SLEDAI score decreased from 11.42+/- 6.74 at baseline to 3.61+/- 2.73 at 6 months (p <0.0001).	TAC was well tolerated at the administered dose, though one patient developed severe lung infection.	2B
A randomized double-blind, placebo-controlled trial of allogeneic umbilical cord-derived mesenchymal stem cell for lupus nephritis Deng et al. (55) Study type: Randomized double-blind, placebo-controlled trial Type of LN: WHO class III or IV	Adult study including some JSLE patients Total: 18 F 17 M 1 Age, years, mean (SD) Placebo: 29 (7) hUC-MSC: 29 (10)	Allogeneic hUC-MSC or placebo. Induction therapy: IV methylprednisolone at the discretion of the investigator plus low-dose IV CYP six pulses at a fixed dose of 500 mg every 2 weeks. After the first 11 patients were treated, the induction CYP was changed to a rescue treatment [i.e., the investigator considered initiating CYP 4 weeks after commencing the study treatment (hUC-MSC or placebo)]. Earlier initiation of CYP was however still permitted (investigators discretion).	Primary outcome: Remission of nephritis (combined partial and complete remission defined as stabilization or improvement in renal function, urinary red blood cells <10 per high power field and reduction of proteinuria <3 g/day if baseline proteinuria >3 g/day or at least a 50% reduction in proteinuria or <1g/day if baseline proteinuria was in the sub nephrotic range.	Remission of nephritis occurred in 9 of 12 patients (75%) in the hUC-MSC group and 5 of 6 patients (83%) in the placebo group (no p value stated). A similar proportion of patients in each treatment arm achieved complete remission (no p value stated). Improvements in serum albumin, complement, renal function, SLEDAI, BILAG were similar in both groups (no p value stated). The trial was abandoned after 18 patients were enrolled, clear it would not demonstrate a positive treatment effect.	One patient on placebo had a stroke and another had ascites. One patient on hUC-MSC had leucopenia, pneumonia and subcutaneous abscess and another died of severe pneumonia.	1b
		Maintenance therapy: prednisolone and MMF.	Stabilization of renal function was defined as change in serum creatinine concentration of <20% compared with baseline concentration and improvement in renal function was defined as a reduction in serum creatinine of at least 20% compared with baseline. Complete response was similarly defined except for reduction of proteinuria <0.3g/day.	Secondary endpoints: improvement in lupus activity scores (SLEDAI and BILAG), complement concentration, anti-dsDNA antibody and ANA titres, death and commencement of permanent dialysis or renal transplantation.		

*LN, lupus nephritis; M, Male; F- female; MMF, mycophenolate mofetil; PCR, protein creatinine ratio; ESRD, End stage renal disease; BILAG, British Isle Lupus Assessment; ACE, angiotensin converting enzyme; ARB, angiotensin receptor blocker; TAC, tacrolimus; CYC, cyclophosphamide; ELNT, Euro Lupus Nephritis treatment regimen; BILAG, British Isle Lupus Assessment; NSAID, nonsteroidal anti-inflammatory drugs; TAC, tacrolimus; MZR- mizoribine; AZA, azathioprine; ECLAM, European Consensus Lupus Activity Measurement; TAC, tacrolimus; SLEDAI score, Systemic Lupus Erythematosus Disease Activity Index scores; hUC-MSC, human umbilical cord-derive mesenchymal stem cell; UPC ratio, urine protein creatinine ratio*.

A small Chinese study including 70 JSLE and adult SLE patients (all >16-years old, mean age of the study population not specified) has compared maintenance tacrolimus and azathioprine treatment, showing similar, low LN relapse rates in both treatment arms, with tacrolimus demonstrating a more favorable safety profile than azathioprine (53). In another small study from China, 26 patients with LN and persistent proteinuria of >1.5g/24-h despite treatment with cyclophosphamide (>8 g in <6-months), were commenced on 2–3mg of tacrolimus daily. 23/26 patients demonstrated an overall renal response (10 complete and 16 partial renal response). Most patients had biopsy confirmed LN (class III=5, class IV=2, class V=5, class III+V=7, class IV+V=4 and unknown *n* = 3), with patients with class V LN demonstrating higher rates of remission (Table 4) (54). Further research is required in JSLE to evaluate the role of tacrolimus in studies that are sufficiently powered.

### Stem Cell Treatment

In a small randomized controlled trial including 18 JSLE and SLE patients (mean age 29-years) with WHO class III/IV LN, no additional effect was seen in those treated with human umbilical cord-derived mesenchymal stem cells over and above standard immunosuppression (intravenous methylprednisolone and cyclophosphamide followed by maintenance oral prednisolone and MMF, Table 4) (55). The trial was stopped early when it became clear that it would not demonstrate a positive treatment effect.

#### New Evidence Relating to the Specific SHARE LN Treatment Recommendations

New evidence relating specifically to each of the SHARE LN recommendations is very limited. Where new evidence could be identified from pediatric, young adult or adult SLE studies it is summarized below.

SHARE recommends that “*Immunosuppressive treatment should be guided by a diagnostic renal biopsy”* (2). No new original research studies could be identified that relate to this. However, very similar statements have also been endorsed by the Joint EULAR and European Renal Association European Dialysis and Transplant Association (ERA-EDTA) recommendations for the Management of Adult and Pediatric Lupus Nephritis (65), and the American College of Rheumatology (ACR) Guidelines for Screening, Treatment, and Management of Lupus Nephritis (66).

When assessing response to initial LN induction treatment, SHARE recommends that “*Partial renal response should be achieved preferably by 6 months but no later than 12 months following initiation of treatment”* and that “*Treatment should aim for complete renal response with urine protein:creatine ratio*<*50 mg/mmol and normal or near-normal renal function (within 10% of normal GFR)”* (2). Again, there is no new pediatric evidence relating to this recommendation. An adult SLE study has since suggested that partial renal response should be achieved sooner (by 12-weeks after commencement of induction therapy for class III or IV LN), with lack of a partial renal response by 12-weeks ultimately predicting poor renal response, and damage accrual (67). These authors have also shown that early achievement of a complete renal response (by 12-weeks) is significantly associated with maintaining a complete response at 3-years (*p* = 0.012), less frequent SLE flares (*p* = 0.026) and damage (*p* = 0.029) during the subsequent 10-years of follow-up (68), highlighting the need for assessment for and importance of timely achievement of partial and complete renal response. Further studies are needed to investigate the achievability and impact of such renal outcomes in JSLE.

The SHARE recommendations advocate that “*In case of LN with proteinuria, ACE-inhibitors or ARBs should be considered as additional treatment. Combined use of ACE inhibitors and ARBs should be guided by pediatric nephrologists”* (2). No new original evidence relating to this recommendation could be found. However, the Brazilian Society of Rheumatology consensus guidelines for the diagnosis, management and treatment of LN in adult SLE have also concluded that “*ARBs and ACE inhibitors should be used as antiproteinuric agents unless contraindicated”* (69).

In relation to the treatment of class I LN, SHARE recommended that “*Low-dose prednisone (*<*0.5 mg/kg/day) could be considered in class I LN, although treatment choice should be guided mainly by other clinical features”* and that “*For the treatment of class I LN alone, adding a DMARD is not necessary”* (2). This is echoed by the pediatric Kidney Disease: Improving Global Outcomes (KDIGO) Glomerulonephritis Work Group clinical practice guideline for glomerulonephritis that were published in 2012 (subsequent to the original SHARE literature review), suggesting that patients with class I LN should be treated according to their extrarenal JSLE manifestations (70). For the treatment of class II LN, SHARE made the following recommendations: “*First line treatment of class II LN should be prednisolone (with a starting dose of 0.25–0.5 mg/kg/day, with a maximum of 30 mg/day) tapering over a total duration of 3–6 months”* and “*For the treatment of active class II LN, a DMARD is necessary in persistent proteinuria and/or when failing to taper corticosteroids after 3-months of low dose prednisolone”* (2). Unfortunately, no new evidence could be found in relation to these recommendations.

For induction treatment for class III/IV LN, with or without class V, SHARE recommended “*MMF or intravenous CYC, in combination with corticosteroids”* (2). This is supported by the recent observational study from the UK JSLE Cohort Study (discussed above) which showed comparability between MMF and cyclophosphamide as induction treatments in JSLE (56). From the adult SLE literature, a large randomized trial (*n* = 362, mean age 31.9-years) has demonstrated improved rates of complete and partial renal remission at 24-weeks in patients treated with low-dose MMF, tacrolimus, and steroids compared to monthly intravenous cyclophosphamide and steroids for proliferative LN induction treatment (71).

The SHARE recommendations also advised that “*maintenance treatment for class III or IV LN should consist of MMF or Azathioprine, for at least 3-years*” (2). No new pediatric evidence could be found relating to maintenance therapy. However, the American College of Rheumatology also recommends MMF or Azathioprine for maintenance treatment (in addition to low-dose prednisolone) (66), and the pediatric KDIGO guidelines suggest a calcineurin inhibitor can be used for maintenance therapy if a patient is intolerant to MMF or Azathioprine (70). There was no new evidence guiding the length of maintenance treatment for proliferative LN, or on the treatments that should be used for pure class V LN. Adequately powered randomized controlled trials looking at conventional LN induction and maintenance therapies, investigating of the role of calcineurin inhibitors, and looking at treatment of class V LN in isolation are therefore warranted.

Five of the SHARE recommendations relate to treatment of LN flares and refractory disease (2). No new evidence could be found relating to these recommendations. All the new evidence relating to LN SHARE treatment recommendations is summarized in Supplemental Table 1.

#### Evidence Relating to Lupus APS

Despite this extensive literature search, no papers met the inclusion criteria for this section of the review. It is recognized that management of pediatric APS remains challenging due to a lack of large-scale prospective studies, with most treatment recommendations based on adult studies. Hydroxychloroquine is thought to have anti-thrombotic properties (72). In asymptomatic patients with persistently positive antiphospholipid antibodies, the use of low dose aspirin is controversial, with one small placebo-controlled trial showed no benefit after 2-years. For those who have already suffered from a thrombosis, the main goal of treatment is to prevent further thrombosis through treatment with long term anti-coagulation therapy such as warfarin (73). The role of immunosuppressive treatment remains uncertain (71, 72).

#### Further Evidence Relating to the Specific SHARE Treatment Recommendations for Pediatric APS and Pediatric Catastrophic Antiphospholipid Syndrome

A further literature search was performed specifically reviewing for new evidence relating to each SHARE management recommendation for pediatric APS and CAPS. No new evidence could be found relating to the specific management for pediatric APS. A case series of 21 patients with pediatric CAPS (from 1990 to 2013) was found, which suggested that immunosuppression with corticosteroids or rituximab may confer survival benefit. In this study, none of the patients who received rituximab died, however, the odds ratio for survival crossed 1 and was not statistically significant, potentially likely relating to the small sample size (74). Case reports have also suggested ecluzimab may be beneficial in treatment of CAPS in adults (75, 76) however this has not yet been assessed in children.

### Limitations of Novel Data to Inform Treatment Recommendations

This review highlights that treatment paradigms in JSLE are often needing to be extrapolated from adult SLE, whilst RCTs in JSLE are particularly scarce, especially any that are sufficiently powered to demonstrated statistical significance. Most available treatment options are not targeted (conventional DMARDs), and known to cause significant associated adverse events and toxicity, particularly in vulnerable children and young people (40, 77). Although biologic therapies are used extensively for many autoimmune conditions, there have been several notable setbacks in developing a robust evidence base for SLE, with only belimumab so far licensed for use in SLE in the past 50-years (40). Difficulties with definitions and use of outcome measures in SLE clinical trials have contributed to these setbacks. The Belimumab in JSLE (PLUTO trial) summarized above (12) raises important questions about the applicability of adult SLE outcome measures in JSLE. In this trial, the adult SLE primary outcome measure (SRI4) was not met in the pediatric age group, but the pediatric-derived major secondary outcome measure (PRINTO/ACR 30, 50) was achieved. Given the known differences in disease activity, severity and damage demonstrated between pediatric, adolescent, and adult SLE (78, 79), it is important that lessons are learnt from such studies.

Most of the more recent published evidence relate to treatment of LN, with a marked dearth of studies on NP-JSLE and APS. T2T approaches are hoped to offer an opportunity to improve further the clinical management of JSLE patients by using existing treatments in a structured way with the aim of more aggressively controlling disease activity at an early stage, preventing organ damage and improving HRQOL (7, 8). Such approaches are already part of routine clinical care in many areas of adult medicine (e.g., rheumatoid arthritis, hypertension, diabetes) (80), with growing international evidence for the potential role of T2T in JIA in recent years (10, 81, 82). Development and testing of such an approach as part of the TARGET LUPUS research programme is eagerly awaited.

## Conclusion

Despite differences in pathogenesis, phenotype, associated morbidity and mortality rate in JSLE, treatment is largely based on adult-SLE clinical trials. High quality large, randomized control trials are particularly lacking in JSLE, and especially for neuropsychiatric lupus and APS both in pediatric and adult age groups. The approval for belimumab for JSLE is the main significant advance in treatment since the original SHARE recommendations literature searches. Overall, the SHARE recommendations remain an important, evidence-informed resource for the clinical and scientific community. The evidence collated in this review from pediatric and adult SLE, will be considered by JSLE experts when developing protocol driven therapeutic strategies and clinical decision support tools, for use within a JSLE T2T study. Randomized controlled trials in or involving children and young people are required to obtain more accurate data on the effectiveness and long-term safety profiles of the treatments already used, and new potential treatments options in JSLE, to ensure treatment for this patient population is evidence based.

## Author Contributions

KG, PP, and ES made a substantial contribution to the conceptualization, methodology, analysis, and interpretation of the literature for this manuscript. MB assisted with the design of the study and ensuring that the literature search strategies aligned with those of the original SHARE initiative. KG and PP drafted the first manuscript. All authors discussed the results and commented on the manuscript. All authors contributed to the article and approved the submitted version.

## Funding

The study was supported by the UK's Experimental Arthritis Treatment Center for Children (supported by Versus Arthritis, the University of Liverpool and Alder Hey Children's NHS Foundation Trust).

## Conflict of Interest

The authors declare that the research was conducted in the absence of any commercial or financial relationships that could be construed as a potential conflict of interest.

## Publisher's Note

All claims expressed in this article are solely those of the authors and do not necessarily represent those of their affiliated organizations, or those of the publisher, the editors and the reviewers. Any product that may be evaluated in this article, or claim that may be made by its manufacturer, is not guaranteed or endorsed by the publisher.
