# Supplementary material for: What Have We Learnt About the Treatment of Juvenile-Onset Systemic Lupus Erythematous Since Development of the SHARE Recommendations 2012?
Source: Front Pediatr. 2022 Apr 14;10:884634. doi: 10.3389/fped.2022.884634 (PMC9047745; doi:10.3389/fped.2022.884634)
Supplement: Supplementary file 1 [file Table_1.pdf]

Supplementary Table 1 - Additional evidence from specific literature searches relating to individual SHARE recommendations (since 2012)

Study	Patients	Key results of relevance to this review
**More Consistent Antimalarial Intake in First 5 Years of Disease Is Associated with Better Prognosis in Patients with Systemic Lupus Erythematosus.** *Pakchotanon et al,* 2018 Study type: cohort study	Adult study 459 patients	"More consistent" use of antimalarials in the first 5-years following SLE diagnosis (defined as patient reported antimalarial agent use >60% of the time) was associated with reduced risk of SLICC-SDI score defined damage, increased achievement of low disease activity (defined as a clinical-SLEDAI-2K score of ≤2, not including serology) and reduced cumulative glucocorticoid dose after 5-years of follow-up.
**Long-Term Clinical Outcomes in a Cohort of Adults With Childhood-Onset Systemic Lupus Erythematosus.** *Groot et al,* 2019 Study type: cohort study	Adult study 111 childhood onset SLE patients	Hydroxychloroquine monotherapy is associated with absence of SLICC-SDI defined damage (OR 0.16, P = 0.009).
**Mortality, causes of death and influence of medication use in patients with systemic lupus erythematosus vs matched controls.** *Bultink IEM et al, 2021* Study type: cohort study	Adult study 4343 SLE patient 21 780 age and sex match controls	Hydroxychloroquine use is associated with a 45% reduction in the hazards of mortality in adult-SLE. Cumulative glucocorticoid use raised the mortality rate.
**The risk of toxic retinopathy in patients on long-term hydroxychloroquine therapy.** Melles RB et al, 2014 Study type: retrospective case-control study	2361 patients on long term hydroxy-chloroquine therapy	Due to advances in eye screening, hydroxychloroquine retinopathy has been found to be more common than previously thought, with a study showing a prevalence of 7.5% in adult-SLE patients taking hydroxychloroquine for a minimum of 5-years. A total daily dose of >5mg/kg (using actual body weight) was found to be associated with increased risk. Renal impairment and concurrent tamoxifen citrate therapy also increased the risk of retinopathy.
**Hydroxychloroquine and Chloroquine Retinopathy Monitoring Guideline and Recommendations 2020.** *Ophthalmologists RCo.,* 2020.	Adult guideline	The guideline states that when long-term hydroxychloroquine treatment is planned, patients should receive a baseline examination (within 12-months), followed by annual screening from year 5 of treatment onwards. In patients with additional risk factors for retinopathy (e.g., Tamoxifen use, impaired renal function (estimated glomerular filtration rate of <60ml/min/1.73m^2), hydroxychloroquine dose >5mg/kg/day) annual monitoring from baseline is recommended. Despite a lack of evidence in patients <18-years, these guidelines advise that these patients on long term hydroxychloroquine should also be referred for monitoring as per the adult criteria.
**Usefulness of cellular text messaging for improving adherence among adolescents**	Paediatric study	Only 32% of adolescents and young adults with SLE were compliant with hydroxychloroquine.

**and young adults with systemic lupus erythematosus.**<br>*Ting et al,* 2012<br>Study type: cohort	70 patients	
**Medication Nonadherence in Systemic Lupus Erythematosus: A Systematic Review.**<br>*Mehat et al,* 2017<br>Study type: systematic review	Systematic review	43-75% of adult-SLE patients were non-compliant with SLE treatment, with the majority of studies reviewed consistently reported that over half of patients are nonadherent. Studies also showed that a third of patients discontinue therapy after 5 years, with risk factors for nonadherence being depression, rural residence, lower education level, and polypharmacy.
**Hydroxychloroquine in systemic lupus erythematosus: results of a French multicentre controlled trial (PLUS Study).**<br>*Costedoat-Chalumeau et al,* 2013<br>Study type: Randomised controlled trial	Adult study<br><br>573 patients	Tailoring hydroxychloroquine dose to a target therapeutic blood concentration did not reduce the frequency of SLE flares.<br>However, hydroxychloroquine blood levels increased spontaneously between study inclusion and randomisation, suggesting improved adherence to hydroxychloroquine treatment in all patients, likely in response to the information that was sent to patients about the study.
**The Clinical Significance of Monitoring Hydroxychloroquine Levels in Patients with Systemic Lupus Erythematosus: A Systematic Review and Meta-Analysis.**<br>Garg et al, 2020.<br>Study type: systematic review	Systematic review and meta analysis	This study found 3-times higher odds of reported nonadherence in patients with low hydroxychloroquine levels (OR 2.95, 95% CI 1.63, 5.35 p <0.001).
**Improvement of medication adherence in adolescents and young adults with SLE using web-based education with and without a social media intervention, a pilot study.**<br>Scalzi et al, 2018<br>Study type: pilot study	Paediatric study<br><br>37 patients	A web-based education and a social media intervention was shown to significantly improve adherence to medications in adolescents and young adults with SLE (p <0.001).<br>Self-reported medication adherence was significantly higher than objectively measured indices of adherence (e.g. the medication possession ratio), highlighting the need for objective measures of adherence, such as blood levels.<br>Novel approaches such as social media interventions may help empower patients to manage their own medications effectively.
**2019 update of the EULAR recommendations for the management of systemic lupus erythematosus.**<br>*Fanouriakis et al,* 2019<br>Study type: Systematic review	Systematic review	EULAR recommendations for SLE also advise addition of methotrexate, azathioprine, or mycophenolate mofetil (MMF) in patients whose symptoms are not controlled with corticosteroids and hydroxychloroquine.<br>Cyclophosphamide is suggested for severe organ threatening or life-threatening SLE or for patients who do not respond to other immunosuppressive agents.<br>These recommendations advise that belimumab is used for patients with frequent relapses or those not able to taper steroid dose despite the above standard of care.<br>Consideration of rituximab is suggested for organ-threatening disease refractory to standard immunosuppressive agents or where these are contraindicated or not tolerated.

**Rituximab therapy has a rapid and durable response for refractory cytopenia in childhood-onset systemic lupus erythematosus.**<br>*Olfat et al,* 2015<br>Study type: cohort study	Paediatric study<br><br>24 patients	This retrospective cohort study assessed 24 JSLE patients treated with rituximab for refractory cytopenias, 19 of whom had haemolytic anaemia. Overall, 96% of patients showed complete response after the first course of rituximab (defined as Hb >120 g/L for haemolytic anaemia and platelet count >100x10^9/L for patients with thrombocytopenia). The median time to complete response for patients with haemolytic anaemia was 85 days.
**Hematological features of pediatric systemic lupus erythematosus: suggesting management strategies in children.**<br>Gokce et al, 2012<br>Study type: cohort study	Paediatric study<br><br>43 patients	This study showed benefits from rituximab in cases of haemolytic anaemia resistant to steroid and intravenous immunoglobulin (IVIG) treatment.
**The indications, efficacy and adverse events of rituximab in a large cohort of patients with juvenile-onset SLE.**<br>*Watson et al,* 2015<br>Study type: cohort study	Paediatric study<br><br>63 patients	Rituximab use in patients with JSLE over a 10-year period (2003-2013): 63 patients all received a dose of 750mg/m^2/dose approximately 2-weeks apart. This is also the dose as per SHARE recommendation.<br>Study concluded that rituximab improves disease activity in children with lupus and serious adverse events are infrequent.<br>Controlled studies are required.
**Utility and safety of rituximab in pediatric autoimmune and inflammatory CNS disease.**<br>*Dale at al,* 2014<br>Study type; cohort stidy	Paediatric study<br><br>144 patients	144 children with autoimmune and inflammatory disorders of the central nervous system (18/144 with NP-SLE) treated with rituximab demonstrated "definite" clinician-defined improvement with rituximab in 5/18 patients, "probable" in 7/18, "possible" in 5/18 and "no improvement" in 1/18 patients.
**Safety and efficacy of combined cyclophosphamide and rituximab treatment in recalcitrant childhood lupus.**<br>*Ale'ed et al,* 2014<br>Study type:cohort study	Paediatric study<br><br>18 patients	Included 2 cases of lupus cerebritis which improved with combined rituximab and cyclophosphamide treatment.
**Rituximab therapy for severe pediatric systemic lupus erythematosus**<br>*Su et al,* 2012<br>Study type: cohort study	Paediatric study<br><br>20 patients	20 children with SLE reported that in 10/20 (50%) cases, delirium and cognitive disorders improved after one-month of rituximab treatment.
**Mycophenolate mofetil and deflazacort combination in neuropsychiatric lupus: a decade of experience from a tertiary care teaching hospital in southern India.**<br>*Gupta et al,* 2017<br>Study type: cohort study	Adult study<br><br>88 patients	88 adult patients with NP-SLE treated with MMF and deflazacort showed complete response (defined as complete resolution of initial neuropsychiatric signs and symptoms) in 83.9% of patients at 1-year follow up, and in 92.3% of patients at last follow up (median 33-months).

**Lack of partial renal response by 12 weeks after induction therapy predicts poor renal response and systemic damage accrual in lupus nephritis class III or IV.**<br>*Hanaoka et al,* 2017.<br><u>Study type:</u> cohort study	Adult study<br><br>80 patients	This study suggests partial renal response should be achieved sooner (by 12-weeks after commencement of induction therapy for class III or IV LN), with lack of a partial renal response by 12-weeks ultimately predicting poor renal response, and damage accrual.
**Early achievement of complete renal response predicts good long-term renal outcome and low systemic damage in newly diagnosed lupus nephritis class III or IV.**<br>*Hanaoka et al,* 2015<br><u>Study type:</u> cohort study	Adult study<br><br>18 patients	Early achievement of a complete renal response (by 12-weeks) is significantly associated with maintaining a complete response at 3-years (p=0.012), less frequent SLE flares (p=0.026) and damage (p=0.029) during the subsequent 10-years of follow-up.
**Multitarget therapy for induction treatment of lupus nephritis: a randomized trial.**<br>*Liu et al,* 2015<br><u>Study type:</u> randomised trial	Adult study<br><br>362	This large, randomized trial (n=362, mean age 31.9-years) has demonstrated improved rates of complete and partial renal remission at 24-weeks in patients treated with low-dose MMF, tacrolimus, and steroids compared to monthly intravenous cyclophosphamide and steroids for proliferative LN induction treatment.
**American College of Rheumatology guidelines for screening, treatment, and management of lupus nephritis.**<br>*Hahn et al,* 2012<br><u>Study type:</u> Clincial gudielines	Clinical guideline	The American College of Rheumatology also recommends MMF or Azathioprine for maintenance treatment (in addition to low-dose prednisolone) for class III or IV LN.
**Consensus of the Brazilian Society of Rheumatology for the diagnosis, management and treatment of lupus nephritis.**<br>*Klumb et al,* 2015<br><u>Study type:</u> literature review	Adult studies	*"ARBs and ACE inhibitors should be used as antiproteinuric agents unless contraindicated"*
**KDIGO Clinical Practice Guideline for Glomerulonephritis.**<br>*Kidney Disease Improving Global Outcomes (KDIGO*), 2012<br><u>Study type:</u> clinical pratice guideline	Clinical guideline	Patients with class I LN should be treated according to their extrarenal juvenile SLE manifestations.<br><br>A calcineurin inhibitor can be used for maintenance in LN therapy if a patient is intolerant to MMF or Azathioprine.

**Pediatric Catastrophic Antiphospholipid Syndrome: Case Study and Literature Review.**<br>*Defreitas et al*, 2014<br>Study type: case series	Paediatric<br><br>21 patients	Immunosuppression with corticosteroids or rituximab may confer survival benefit. None of the patients who received rituximab died, however, the odds ratio for survival crossed 1 and was not statistically significant, potentially likely relating to the small sample size.
**A case of relapsing and refractory catastrophic anti-phospholipid syndrome successfully managed with eculizumab, a complement 5 inhibitor.**<br>*Wig et al,* 2015<br>Study type: case report	Adult<br><br>1 patient	Ecluzimab may be beneficial in treatment of CAPS in adults
**Brief Report: Induction of sustained remission in recurrent catastrophic antiphospholipid syndrome via inhibition of terminal complement with eculizumab**.<br>*Shapira et al,* 2012<br>Study type: case report	Adult<br><br>1 patient	Ecluzimab may be beneficial in treatment of CAPS in adults

SLE – Systemic Lupus Erythematosus, SLICC-SDI - Systemic Lupus International Collaborating Clinics/American College of Rheumatology Damage Index, SLEDAI score - Systemic Lupus Erythematosus Disease Activity Index scores, JSLE – Juvenile Systemic Lupus Erythematosus, NP-SLE – neuropsychiatric SLE, MMF – mycophenolate mofetil, LN – lupus nephritis, CAPS – Catastrophic antiphospholipid syndrome
